# Biogenic Nanosilver against Multidrug-Resistant Bacteria (MDRB)

**DOI:** 10.3390/antibiotics7030069

**Published:** 2018-08-02

**Authors:** Caio H. N. Barros, Stephanie Fulaz, Danijela Stanisic, Ljubica Tasic

**Affiliations:** Laboratory of Chemical Biology, Institute of Chemistry, State University of Campinas, Campinas 13083-970, Brazil; caionasibarros@gmail.com (C.H.N.B.); ste.fulaz@gmail.com (S.F.); danijela.stanisic@iqm.unicamp.br (D.S.)

**Keywords:** silver nanoparticles, biological synthesis, multidrug-resistant bacteria

## Abstract

Multidrug-resistant bacteria (MDRB) are extremely dangerous and bring a serious threat to health care systems as they can survive an attack from almost any drug. The bacteria’s adaptive way of living with the use of antimicrobials and antibiotics caused them to modify and prevail in hostile conditions by creating resistance to known antibiotics or their combinations. The emergence of nanomaterials as new antimicrobials introduces a new paradigm for antibiotic use in various fields. For example, silver nanoparticles (AgNPs) are the oldest nanomaterial used for bactericide and bacteriostatic purposes. However, for just a few decades these have been produced in a biogenic or bio-based fashion. This review brings the latest reports on biogenic AgNPs in the combat against MDRB. Some antimicrobial mechanisms and possible silver resistance traits acquired by bacteria are also presented. Hopefully, novel AgNPs-containing products might be designed against MDR bacterial infections.

## 1. Introduction

Antimicrobial resistance refers to the evolutionary capacity developed by microorganisms such as bacteria, fungi, viruses, and parasites to fight and neutralize an antimicrobial agent. According to the World Health Organization (WHO) [1], the intensive use and misuse of antimicrobials has led to an expansion of the number and types of resistant organisms. Moreover, the use of sub-therapeutic antibiotic doses to prevent diseases in animal breeding to improve animal growth can select resistant microorganisms, which can possibly disseminate to humans [2].

The number of pathogens presenting multidrug resistance has had an exponential increase in recent times and is considered an important problem for public health [3]. A wide number of bacteria have been reported as multidrug-resistant (MDR), and they present a high cost of management, including medicines, staff capacity, isolation materials [4], and productivity loss [5]. For instance, in the USA, the cost of conventional tuberculosis treatment for the drug-susceptible bacterium is $17,000 and up to $482,000 for the treatment of the MDR bacterium [5]. In 2017, WHO published the first list of antibiotic-resistant pathogens offering risk to human health and, as such, the development of new drugs is crucial. Priority 1 (critical) microorganisms are carbapenem-resistant *Acinetobacter baumannii*; carbapenem-resistant *Pseudomonas aeruginosa*; and carbapenem-resistant, ESBL-producing *Enterobacteriaceae*. Accounting for priority 2 (high) are vancomycin-resistant *Enterococcus faecium*; methicillin-resistant, vancomycin-intermediate and resistant *Staphylococcus aureus*; clarithromycin-resistant *Helicobacter pylori*; fluoroquinolone-resistant *Campylobacter* spp.; fluoroquinolone-resistant *Salmonellae*; and cephalosporin-resistant, fluoroquinolone-resistant *Neisseria gonorrhoeae*. In priority 3 (medium) are penicillin-non-susceptible *Streptococcus pneumoniae*, ampicillin-resistant *Haemophilus influenzae*, and fluoroquinolone-resistant *Shigella* spp. [6].

The use of drugs combinations, two or more antimicrobial drugs to combat MDRB [7], is already employed in cancer therapy [8], HIV-patients [9], and malaria patients [10]. On the other hand, research groups around the globe are suggesting innovative solutions to treat resistant organisms. Xiao et al. [11] synthesized the block copolymer poly (4-piperidine lactone-b-ω-pentadecalactone) with high antibacterial activity against *E. coli* and *S. aureus*, and low toxicity to NIH-3T3 cells, and suggested that cationic block copolymer biomaterials can be employed in medicine and implants. Zoriasatein et al. [12] showed that a derivative peptide from the snake (*Naja naja*) has an antimicrobial effect against *S. aureus*, *B. subtilis*, *E. coli*, and *P. aeruginosa*. Al-Gbouri and Hamzah [13] reported that an alcoholic extract of *Phyllanthus emblica* exhibits antimicrobial activity against *E. coli*, *S. aureus*, and *P. aeruginosa* and it inhibits biofilm formation of *P. aeruginosa*. Naqvi et al. [14] suggested the combined use of biologically synthesized silver nanoparticles (AgNPs) and antibiotics to combat the MDRB.

**Figure 1:**
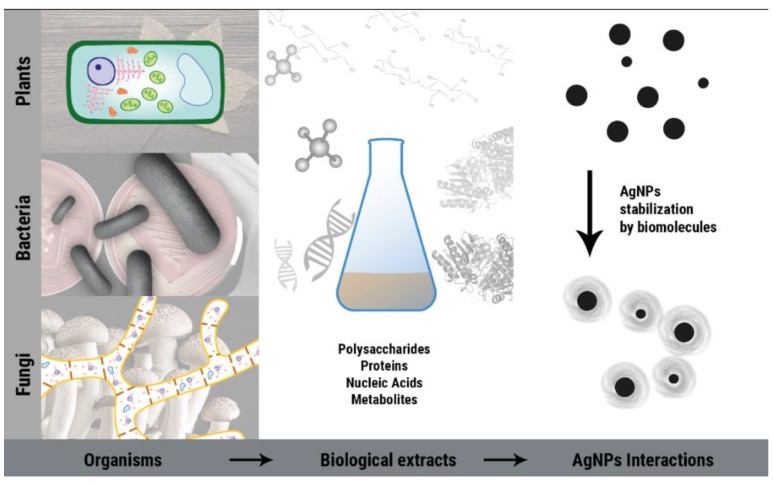
Biological extracts may be prepared from any part of plant material, or via extracellular/intracellular processes using fungi and bacteria cultures. The extracts are rich in biomolecules such as sugars, proteins, nucleic acids, and metabolites that either have a stabilizing potential or reducing and stabilizing potential for the formation of silver nanoparticles.

The increasing utilization and in-depth studies of nanomaterials have brought new perspectives towards new antimicrobial materials and nanocomposites that could add-in to the MDRB pandemic that we are currently facing. Nanoparticles and nanocomposites comprising zinc oxide [15], copper oxide [16], iron oxide [17], and, especially, silver, have been widely used in textiles [18,19], dental care [20], packaging [21], paints [22], and in a whole myriad of applications. Silver nanoparticles are one of the most exploited nanomaterials for this end, as they have been used for over a century in the healing of wounds and burns. Although chemical methods were successfully employed for AgNPs synthesis, with the need to use sustainable and non-toxic methods in chemistry, a biocompatible modality of AgNPs synthesis came about by using biological routes for nanoparticle synthesis (Figure 1). Biosynthesis or bio-based synthesis of AgNPs may occur through three routes: fungal, bacterial, or by plants, for the reduction of Ag^+^ to Ag^0^. The saturation of Ag^0^ monomers in suspension eventually leads to a burst-nucleation process [23] in which nanoclusters of metallic silver are produced and stabilized by biomolecules from the biological extracts.

The demand of products for the combat of MDR bacterial strains such as *Pseudomonas aeruginosa*, methicillin-resistant *Staphylococcus aureus* (MRSA), vancomycin-resistant *Staphylococcus aureus* (VRSA), erythromycin-resistant *Streptococcus pyogenes*, and ampicillin-resistant *Escherichia coli* [24] has led to the design of powerful antimicrobial materials that are reinforced with silver nanoparticles [25]. Today, in medicinal practice, there are wound dressings, contraceptive devices, surgical instruments, bone prostheses, and dental implants which are coated or embedded with nanosilver [26,27,28,29,30,31]. In daily life, consumers may find nanosilver in room sprays, laundry detergents, water purification devices and paints [26,32,33]. In the final part of this review, some of the recent advances in patented technologies containing AgNPs that establish viable grounds for the development of biogenic AgNPs-containing products for MDRB eradication purposes are cited and discussed.

## 2. Antibiotics

**Figure 2:**
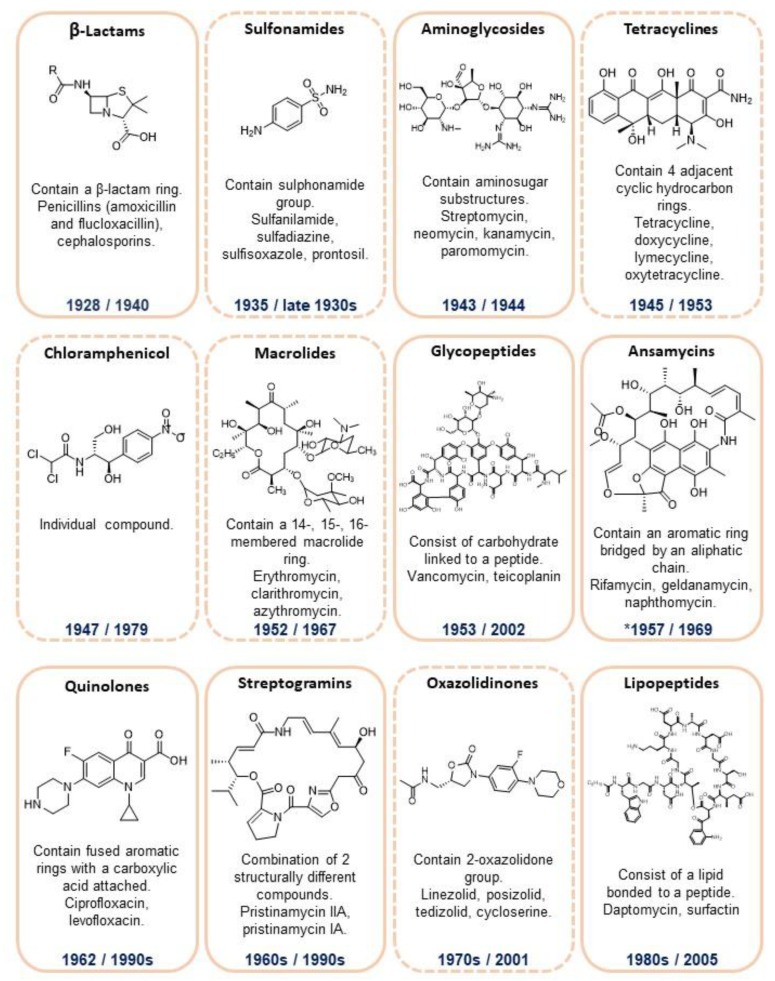
Illustration of different classes of antibiotics. Antibiotics that act as bactericidal agents, i.e., cause cell death, are shown in rectangles with orange borders; antibiotics that act as bacteriostatic agents, i.e., restrict growth and reproduction, are shown in rectangles with dashed line orange borders. Years shown in blue indicate when the antibiotic class was discovered (the first number), and when resistance was first reported (the second number). The structure and years of discovery and resistance refer to the first antibiotic from each class [35,40,41,42,43,44,45,46].

Antibiotics gained popularity because of their effectiveness or activities against microorganisms, as described by Selman Waksman [34], and refers to an application, and not a class of compound or its function [35]. The first compound with antibacterial activity discovered was arsphenamine, synthesized in 1907 by Alfred Bertheim in Paul Ehrlich’s laboratory, with antisyphilitic activity identified in 1909 by Sahachiro Hata [36,37]. Classically, the golden era of antibiotics refers to the period between the 50s and 70s, when the discovery of different classes of antibiotics took place [38]. For a more detailed review of antibiotics and antibacterial drugs, see Bbosa et al., 2014 [39]. Figure 2 illustrates the main antibiotic classes and examples of compounds, with corresponding dates of discovery and resistance as first reported.

## 3. The Emerging of Antimicrobial Resistance

One of the most famous antibiotics, Penicillin, was discovered in 1928 by Alexander Fleming. In 1940, before its public use, the same group identified a bacterial penicillinase [47], an enzyme able to degrade penicillin. This fact can now be related to the number of antibiotic genes that are naturally present in microbial populations [48]. In Japan, during the 50s, genetically transferable antibiotic resistance was identified. This discovery introduced the concept that antibiotic genes could spread among a population of bacterial pathogens using bacterial conjugation [49,50]. This horizontal gene transfer is important throughout genome evolution and currently presents a serious threat [51]. The bacterial genetic elasticity prompts the acquisition of genetic material, mutational adaptations, or changes in gene expression, leading to the survival of the fittest organism and the generation of resistance to antibiotics [52]. For more details regarding antibiotic resistance development, mechanisms, emergence, and spread see further references [52,53,54,55,56,57,58].

Currently, we face a deficiency in the development of new antibiotics to face the growing antimicrobial resistance. The constant increment in the emergence of resistant strains has not been balanced by the availability of new therapeutic agents for many reasons [59,60]. Firstly, policy-makers want to avoid the use of new antibiotics until they are indispensable, because of the resistance development. On the other hand, society needs the pharmaceutical industry to design and develop new drugs, which should not be used. Moreover, antibiotics are used in the short-term, which does not help companies to make a sustained profit. Also, the excessive cost of development and the regulatory onus makes it difficult to attend a demand for cheap antibiotics [61]. Looking at this alarming scenario the design of new therapeutics and/or new approaches is imperative.

## 4. Biogenic AgNPs as a Weapon against Multidrug-Resistant Bacteria (MDRB)

Traditionally, the synthesis of AgNPs using chemical approaches has been the most explored for a better size and shape control, preparation of nanocomposites and elucidation of electronic properties. However, the necessity of applying the well-known antibacterial activity of AgNPs in biological systems propelled the development of a new synthesis approach. The biological, biogenic, or bio-based methods for AgNPs synthesis present four main advantages: (1) increased biocompatibility, once AgNPs are produced in water and capped with biomolecules such as proteins, sugars or metabolites; (2) diminished toxicity, as the reducing agents are natural compounds that usually have mild reducing strength; (3) easy production, such as preparation of an extract from fungi, bacteria or plants, followed by the addition of a silver salt (typically, silver nitrate); and (4) low cost [62]. Despite positive aspects, the lack of control of shape and size of the nanoparticles is still a challenge for biogenic synthesis methods.

Because every biological synthesis is different from another as a consequence of using distinct species, the capping agents on the surface of the nanoparticles may differ. The concept of “protein corona” [63], for instance, describes the existence and dynamics of a protein shell surrounding nanoparticles in a biological environment or after a biological synthesis [64]. The interaction of biologically synthesized AgNPs with a bacterial cell will inherently involve the contact with the microorganism and the outer biomolecule shell. Thus, this interaction is unique as new joint effects (between biomolecules and the silver itself) can arise and improve the antibacterial action due to a change in toxicity, cell uptake, and bio-distribution [65].

In the case of MDRB, the mechanism of action of AgNPs is distinct from the mechanism by which traditional antibiotics act, and thus resistance does not pose an obstacle that cannot be overcome in most cases. In the following sections, each type of biological synthesis is detailed along with a literature review of biogenic AgNPs being used against MDRB. In most of the papers reviewed, the bacterial strains used for susceptibility and antibacterial tests were clinical isolates from hospital patients, however the list of antibiotics to which the strain is resistant is not always described. Also, in many cases the strain used is standardized (ATCC strains, for example), but no details on the drug resistance capacity are provided. Here, we emphasize the examples where the provenience and description of the bacterial strain are well detailed, along with a robust antibacterial testing methodology.

### 4.1. Fungal AgNPs against MDRB

The synthesis of AgNPs using fungal cells may be performed outside the cells (extracellular synthesis) or inside the cells (intracellular synthesis) [66]. The former is the most recurrent in the literature, in which a fungal filtrate is obtained after the cultivation of the microorganism and a silver salt solution is added to it. Advantages of extracellular synthesis include ease of purification (as nanoparticles are not inside or attached to the fungus), facilitated downstream processing, and improved size control [67]. Despite usually having high reproducibility, fungal syntheses are time-consuming, as the fungi grow at a slower rate when compared to bacteria or the preparation of a plant extract. Moreover, the reduction of silver ions is also a gradual process, taking up to 96 h for completion. *Fusarium oxysporum* is perhaps the most studied species for AgNPs biosynthesis [19,68]; the mechanism of nanoparticle formation involves the reduction of silver(I) by a nitrate reductase and a shuttle quinone [69]. Scandorieiro et al. [70] demonstrated the synergistic effect of *F. oxysporum* produced AgNPs with oregano essential oil against a range of antibiotic-resistant bacterial strains, including MRSA and beta-lactamase producing strains. Naqvi et al. [14] also showed the effectiveness of a synergistic approach by combining *Aspergillus flavus* produced AgNPs with well-known commercial antibiotics resulting in an increase of up to 7-fold in the area of inhibition against bacterial strains resistant to the same antibiotics. In fact, a combinational therapy is highly desirable taking into consideration the development of AgNPs tolerance in bacteria via genetic evolution [71]. Chowdhury et al. studied the effect of AgNPs synthesized by *Macrophomina phaseolina* against ampicillin and chloramphenicol resistant *E. coli* and noted plasmid fragmentation and a decrease of supercoiled plasmid content upon incubation of the circular DNA with nanoparticles [72]. On the other hand, nanoparticle attachment to the cell wall and leakage of cell components induced by *Penicillium polinicum*-produced AgNPs were observed in transmission micrographs by Neethu et al. [73], which confirms that more than one antibacterial mechanism is possible (this theme is further explored in Section 4.4). Table 1 brings a summary of fungal AgNPs and their activity against MDR bacterial strains.

### 4.2. Bacterial AgNPs against MDRB

Similarly to fungal biosynthesis, bacterial AgNPs biosynthesis may also be performed extra- or intracellularly [82]. The former can be done by using the cell biomass, where the reducing agents are secreted by the cells and the nanoparticles formed might be attached to the bacterial wall (which can possibly extend the purification process). In contrast, using a bacterial supernatant/cell-free extract has the advantage of facilitating the downstream process and purification procedures by utilizing a sterile biomolecule-rich mixture to synthesize the nanoparticles, often with the aid of microwave [83] or light irradiation [84]. Conversely, the intracellular AgNPs synthesis takes place inside the cell, often in the periplasmic space [85]. This mechanism requires a certain metal resistance from the bacteria [86] or exposure to very low concentrations of the silver salt, as the Ag^+^ ion must be imported without causing any major damage. The biggest disadvantage of this method is the purification as the nanoparticles must be removed from the interior of the cells. Ultrasonication is usually the most common method used for this end [87].

Singh et al. [88] prepared AgNPs from the culture supernatant of *Aeromonas* sp. THG-FG1.2 extracted from soil and obtained inhibition of several bacterial strains otherwise completely insensitive to erythromycin, lincomycin, novobiocin, penicillin G, vancomycin, and oleandomycin. Desai et al. [89] reported a hydrothermal biosynthesis of AgNPs using a cell-free extract of *Streptomyces* sp. GUT 21 by autoclaving the bacterial extract along with a silver salt solution. The nanoparticles were between 20–50 nm in size and active towards MDRB up to a concentration of 10 µg mL^−1^. Sunlight exposure is also a good methodology for AgNPs biosynthesis, as demonstrated by Manikprabhu et al. [90]. Nanoparticles were produced from *Sinomonas mesophila* MPKL 26 cell supernatant in contact with silver nitrate upon up to 20 min of sun exposure. Specific secreted extracellular compounds can also be used for AgNPs synthesis. Santos et al. [91] attribute the formation of AgNPs smaller than 10 nm to xanthan gum produced during the growth of *Xanthomonas* spp. The nanoparticles could inhibit, to a certain extent, the growth of MDR *Acinetobacter baumannii* and *Pseudomonas aeruginosa*. Table 2 brings a summary of AgNPs produced by bacteria with activity against MDRB.

### 4.3. AgNPs from Plants against MDRB

Production of AgNPs using plant extracts is perhaps the most explored method in biogenic synthesis, probably due to the easiness of the procedure and wide availability of species to work with [101]. The whole plant, the stem, pod, seeds, fruit, flowers, and, most frequently, leaves are used to prepare an extract, which may be done in cold or hot solvent and almost always utilizes water (despite the fact that organic solvent extracts have also been used). The abundance of components such as reducing sugars, ascorbic acid [102], citric acid [103], alkaloids and amino acids [104], along with slightly soluble terpenoids [105], flavonoids [106], and other metabolites in various parts of the plant may easily act as reducing agents, converting Ag^+^ to AgNPs in shorter times (when compared to fungal or bacterial syntheses). Due to the lower protein content in most plants, the capping biomolecule shell often has a significant contribution of polysaccharides [107] and other molecules. Most reports on plant biosynthesis are studies of plant species found in the surroundings of the university or city where the laboratory is located, however, in vitro-derived culture of plants can also be used for these purposes [108].

Ma et al. [107] reported on the biosynthesis of 60 nm AgNPs using polysaccharide-rich root extract of *Astragalus membranaceus* and compared the bacterial inhibition against reference strains of *E. coli*, *P. aeruginosa*, *S. aureus*, and *S. epidermidis* with clinically isolated MDR strains of these bacteria. Interestingly, the nanoparticles were slightly more active toward the resistant strains.

The nanoparticle size is known to play an important role in antibacterial activity [24], and this is no different for MDR strains. AgNPs synthesized by *Caesalpinia coriaria* leaf extract, which were 50–53 nm were shown to be more active towards MDR bacterial clinical isolates when compared to 79–99 nm AgNPs [109].

Despite the common belief that biological synthesis implies a lack of control for Ag^+^ reduction and poor shape control, Jinu et al. [110] demonstrated the synthesis of cubic and triangular shaped 20 nm AgNPs using *Solanum nigrum* leaf extract. The nanoparticles had a contributing effect along with the antimicrobial plant extract towards six MDRB strains. Moreover, these AgNPs showed antibiofilm activity against *P. aeruginosa* and *S. epidermidis*. Prasannaraj et al. [111] reported an extensive study using ten different plant species for AgNPs biosynthesis, yielding spherical, cubic, and fiber-like nanoparticles. All of them inhibited bacterial growth of clinically isolated MDR pathogens and some also displayed antibiofilm activity against *P. aeruginosa* and *S. epidermidis*. The authors correlate the results with the 3 to 4-fold increase in reactive oxygen species (ROS) by AgNPs.

Intracellular ROS production was also observed by flow cytometry for *Ocimum gratissimum* leaf extract-produced AgNPs [112]; the authors suggest that the membrane damage caused by the nanoparticles could prevent efficient electronic transport in the respiratory chain. This was confirmed by micrographs of MDR *E. coli* and *S. aureus* cells treated with AgNPs, which showed leakage of intracellular content and pits in the membrane.

The antibacterial properties of silver can also be delivered by silver chloride nanoparticles (AgCl-NPs), as shown by Gopinath et al. [113]. AgNPs and AgCl-NPs were produced from *Cissus quadrangularis* leaf extract and were active towards both Gram-negative and Gram-positive MDR strains. In this case, chloride ions were identified in the extract and attributed to the formation of AgCl nanocrystals.

Table 3 presents the gathered data on plant biosynthesis of AgNPs with the corresponding activity against MDRB.

### 4.4. Modes of Action of AgNPs against Bacteria

As stated in previous reviews on the subject [24,133,134,135,136,137], the antibacterial action of silver nanoparticles involves a complex mechanism in which more than one factor can act simultaneously to contribute to an overall effect. Moreover, one must consider the existence of more than one silver species, these being the Ag^0^ in the form of nanoparticles and the Ag^+^ which is released from the surface of the nanoparticles as they are slowly oxidized.

Proteomic analysis of *E. coli* proteins expressed after exposure to AgNPs and Ag^+^ revealed that both have a similar mode of action, such as overexpressing envelope and heat shock proteins. However, the nanoparticles were effective at inhibiting bacteria in the nanomolar concentration, whereas the Ag^+^ ions were effective only in the micromolar range [138]. On the other hand, further reports point to the opposite direction. Ag^+^ release depends on oxidation of metallic silver by oxygen in the air; in a study where *E. coli* was exposed to AgNPs in anaerobic conditions, no bactericidal activity was observed, while in aerobic conditions the usual antimicrobial activity was noticed [139]. This effect can be partially explained by a strong interaction of Ag^+^ with the cell membrane and cell wall components such as proteins, phospholipids, and thiol-containing groups, as well as by a proton leakage that can induce cell disintegration [140]. As much as the affinity of Ag^+^ for thiol groups has been known for decades [141], just recently Liao et al. [142] demonstrated how Ag^+^ can deplete intracellular thiol content of *S. aureus* and bind to cysteine residues of thioredoxin reductase’s catalytic site. This enzyme is one of the most important ones related to the antioxidant mechanism and reactive oxygen species (ROS) levels regulation in bacteria. Binding to respiratory chain enzymes is also a factor for intracellular ROS increase [143]. It is worth noting, however, that the protein corona that involves AgNPs has a significant effect on silver ions release. According to a study performed by Wen et al. [144], the binding of cytoskeletal proteins to AgNPs led to a decrease in Ag^+^ leakage, which could suggest that, similarly, biogenic AgNPs that are capped by biomolecules also have a diminished Ag^+^ release and thus their antimicrobial action would rely much less on this species.

Regarding the action of the nanoparticles, their size, shape and capping molecules may play significant roles when binding to the cell wall, membrane, and their internalization. In a study performed with silver nanospheres, nanocubes, and nanowires, the latter resulted in diminished antimicrobial activity when compared to the first two due to a smaller effective contact area with the cell membrane [145]. The same explanation applies for truncated octahedral AgNPs outperforming spherical AgNPs [146]. Truncated triangular shaped AgNPs had a better performance than all the other shapes in a study conducted against *E. coli* [147]. Acharya et al. [148] recently reported a study on silver nanospheres and silver nanorods acting against *K. pneumoniae* and attributed the antibacterial activity to the {111} plane shapes, which contain the highest atomic density. Smaller sizes of nanoparticles also lead to an enhanced bactericidal effect [149,150]. This effect is due to a greater surface area in contact with the bacteria that facilitate membrane rupture and internalization [151].

**Figure 3:**
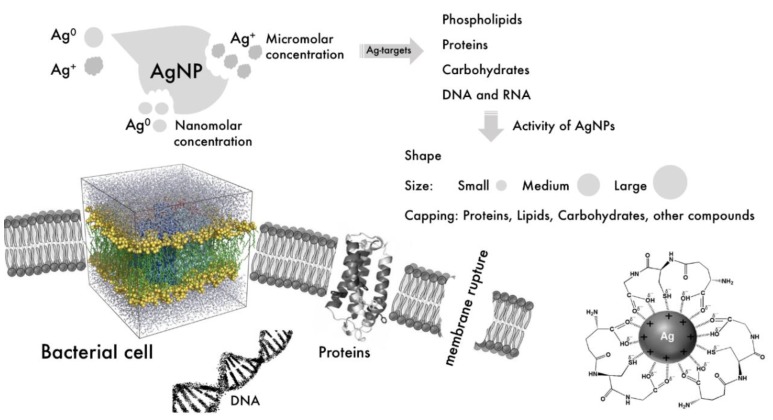
Summary of the factors affecting the antimicrobial capacity of AgNPs and main antibacterial mechanisms. Size, shape and capping agents have a significant influence on the activity against bacterial cells, which are susceptible to nanoparticles because of a strong affinity of the metal with the cell wall and membrane, as well as due to interference in the respiratory chain and generation of reactive oxygen species (ROS).

Perhaps one of the most accepted antibacterial mechanisms involves the association of nanoparticles with the cell wall followed by the formation of “pits” [152] and leakage of cellular contents [153]. This corroborates with the fact that AgNPs are usually more active towards Gram-negative bacteria [154], as Gram-positive bacteria have a thicker peptidoglycan cell wall, which could act as an additional physical barrier. Once inside the bacterial cell (a process that is facilitated by sizes smaller than 5 nm [155]), small nanoparticles are able to interfere with the respiratory chain dehydrogenases [156] and also induce generation of intracellular ROS [112,157], which have the ability to cleave DNA [158] and diminish bacterial life. It must be also pointed out that the interaction of AgNPs with the media which they are suspended in has a great influence on AgNPs physicochemical properties and their action on bacterial cells [159]. Figure 3 illustrates all the major mechanisms by which AgNPs display their antibacterial action.

### 4.5. Bacterial Resistance to Silver

**Figure 4:**
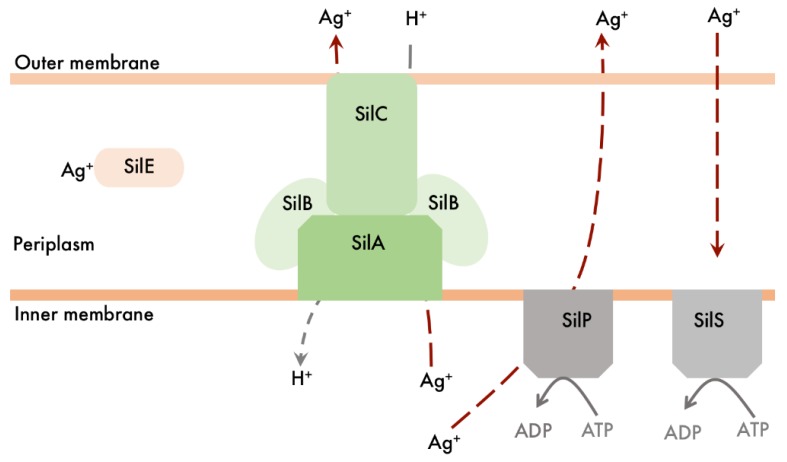
Silver efflux system found in Gram-negative silver-resistant bacteria. SilE is a periplasmic, histidine-rich Ag^+^ binding protein; SilS belongs to a two-component (SilRS) transcription regulation system; SilA, SilB, and SilC comprise a three-component chemiosmotic bacterial proton/cation antiporter.

The increasing application of silver nanomaterials in dressings, packages, and textiles has raised concerns about the development of bacterial resistance to nanosilver, despite the good performance of AgNPs against a range of bacterial strains, as already described. In fact, one of the first reports on resistance to silver was published in 1975, when a strain of *Salmonella typhimurium* resistant to silver nitrate, mercuric chloride, and a range of common antibiotics was identified in three patients in a burn unit [160]. Decades later, this exogenous type of resistance was unveiled by Gupta et al. [161] through the isolation of the plasmid pMG101. This plasmid was identified as the carrier of a silver resistance gene *silE*, which encodes a 143-amino-acid periplasmic Ag^+^-specific protein. Upstream of *silE*, a series of genes from the Sil system encode silver efflux-related proteins, such as a protein/cation antiporter system and a P-type cation ATPase (Figure 4). Resistance to silver attributed to *sil* genes was also recently reported for clinical isolates of *Klebsiella pneumonia* and *Enterobacter cloacae* [162]*.* Endogenous (mutational) silver resistance may also be observed, as reported by Li et al. [163], who observed silver resistance induced in *E. coli* cells by selectively culturing bacterial cells in increasing concentrations of silver nitrate. In this case, mutant cells were deficient in major porins (OmpF and OmpC). Silver efflux is also mediated through a CusCFBA efflux pump system, which has a high amino acid sequence similarity with the Sil system, in spite of being an endogenous type of resistance [164]. Crystal structures of proteins of the CusCFBA system suggest a methionine shuttle efflux mechanism, in which Ag^+^ ions are ejected from the bacterial periplasm [165,166]. Nuclear magnetic resonance (NMR) and inductively coupled plasma mass spectrometry (ICP-MS) studies have demonstrated that silver ions may induce a histidine kinase (CuS) dimerization and this conformational change may have a reflex on the upregulation of genes encoding the CusCFBA transport system [167]. The *E. coli* gene *ybdE* belonging to the K38 chromosome was also pointed out as related exclusively to Ag^+^ resistance since its deletion in silver-resistant mutant strains had no effect on Cu^+^ resistance [168]. Graves et al. [71] recently performed an extensive study using a non-resistant *E. coli* strain for an evolutionary analysis focused on mutations acquired upon exposure to silver nitrate and silver nanoparticles. After 300 generations, the Minimum Inhibitory Concentration (MIC) (using more than one type of AgNPs) of treated bacteria was already between 1.40 and 4.70 times the MIC of control bacteria. Three main mutations were observed: (1) in the *cuS* gene, which encodes the already mentioned histidine kinase which functions as a sensor for the CusCFBA efflux pump; (2) in the *purL* gene, which encodes for an enzyme involved in *de novo* purine nucleotide biosynthesis; and (3) in the *rpoB* gene, responsible for an RNA polymerase beta subunit.

It is worth noting, however, that most of the studies cited are related to exogenous and endogenous Ag^+^ resistance. The release of Ag^+^ ions by AgNPs is only one of the forms by which AgNPs might be antimicrobial, as explained in Section 4.4. Few studies have looked at resistance to silver nanoparticles. For instance, Panacek et al. [169] have observed *E. coli* resistance to 28 nm AgNPs in sub-MIC concentrations without any genetic changes noted in *E. coli*. Only a phenotypic change in production of flagellin was noted. Flagellin, an adhesive protein of the flagellum, related to biofilm formation and motility, was found to readily induce nanoparticle aggregation and attenuate their antimicrobial capacity. There is still much to be researched and discovered on outer membrane–metal interactions, especially what accounts for different capping agents, topography, and morphology of AgNPs. Also, other bacterial species and strains must be studied as to map genetic and/or phenotype modifications induced by AgNPs.

## 5. Nanosilver Applications in Antimicrobial Products

The well-documented antimicrobial activities of AgNPs have attracted great attention from researchers and companies and caused manufacturing of many products which are in everyday use. For instance, dressings, biomedical equipment, paints, packaging materials, and gels containing nanosilver formulations are widely used. However, the number of AgNPs-containing products that are focused on or have been tested against MDRB is still unexpressive and modest. This is even surprisingly true when it comes to biogenically or bio-based synthesized AgNPs. Nevertheless, among many patents of products containing nanosilver, there are some possible applications of patented formulations in the combat against resistant bacteria, which are summarized in Table 4.

Despite the controversy that involves the oral use of silver nanoparticles, a recent patent has established a preparation involving AgNPs active towards MDRB suggesting many possible forms of administration, including oral, topical, and intravenous [171]. An invention communicated by Holladay et al. [170] postulates compositions containing AgNPs that may be introduced into a hydrogel for the treatment of various types of infections and inflammations, with activity against MDR *E. cloacae*, *K. pneumoniae*, *E. coli*, *P. aeruginosa*, and *A. Acinetobacter*. In fact, the well-known wound healing capacity of nanosilver is often exploited in dressings and plasters. Liang et al. [178] developed an AgNPs/chitosan composite with amphiphilic properties—a hydrophobic and waterproof surface and a hydrophilic one with a capacity to interact with water and inhibit the growth of the drugs resistant *S. aureus*, *E. coli*, and *P. aeruginosa*. It is important to point out that these types of dressings with asymmetric wettability properties also enhance re-epithelization and collagen deposition and might be very helpful for wound healing not just because of their antiseptic properties.

Nanocrystalline silver coatings are already available commercially, for example, ACTICOAT™ has been used against MDR *P. aeruginosa* in burn wound infections in rat models [179]. This dressing has also been proven to be effective against methicillin-resistant *S. aureus*, by inhibiting bacterial growth in burn wounds. But it also decreases the secretion and swelling of the damaged tissue areas [180], which speeds up processes of wound healing.

An invention deposited by Paknikar (2006) [176] claims the production of biologically stabilized AgNPs, which were produced from various plants parts, and their incorporation into a variety of possible carriers, such as ointments, sprays, membranes, plasters. The nanoparticles were shown to successfully inhibit MDR strains of *P. aeruginosa* and other highly resistant bacterial strains: *E. coli* ATCC 117, *P. aeruginosa* ATCC 9027, *S. abony* NCTC 6017, *S. typhimurium* ATCC 23564, *K. aerogenes* ATCC 1950, *P. vulgaris* NCBI 4157, *S. aureus* ATCC 6538P, *B. subtilis* ATCC 6633., and *C. albicans*, and, interestingly, were non-cytotoxic towards human leukemic cells (K562), carcinoma cells (HEPG2), and mouse fibroblasts (L929) in the concentrations used against cited MRDB.

Also, there are some reports on materials that contain AgNPs, such as a multipurpose nanocomposite comprising silver nanotriangles and silicon dioxide, which was developed and tested against vancomycin-resistant bacteria *E. Faecalis* (ATCC 51299) [173]. There is a nanocomposite of silver and silver oxide active towards methicillin-resistant *S. aureus* and a broad spectrum of pathogenic bacteria associated with common infections and inflammations in humans [177].

Common household objects can also be enriched with AgNPs to enhance their antimicrobial potential; for example, nanosilver has been used as a detergent additive to enhance the antibiotic effect of the surfactant while not inducing any decrease in the detergent capability of a product [174]. The detergent can be used to disinfect resistant *E. coli* strains. Enhanced hygiene and diminished contamination were also achieved by reinforcing aprons with AgNPs; the material was successful in inhibiting methicillin-resistant *S. aureus*. Cheng and Yan [172] reported and patented the invention on antimicrobial plant fibers enriched with AgNPs that showed strong antimicrobial activity. This material may be applied in various types of linings, clothing, and even for fabricating laboratory or medical coats with improved disinfection properties and thus avoid bacterial contamination.

As stated, there are still much to be discovered and researched until novel fabrics, commodities, and/or pharmaceuticals based on biogenic or bio-based silver nanoparticles became suitable for everyday applications.

## 6. Conclusions

Some of the main reasons for observing the multidrug resistance in bacteria were discussed along with an introduction of biogenic silver nanoparticles as an alternative or combined technology to overcome this growing health problem. Even though bio-based silver containing nanomaterials are usually not ingested as known antibiotics, mainly due to a lack of understanding of the nanotoxicology associated with nanosilver in the bloodstream or in organs, AgNPs may be incorporated in products such as dressings, sprays, textiles, and paints for MDRB combat to a certain extent. Topical use of ointments and wound dressings have become quite common, as AgNPs not only inhibit bacteria growth but also stimulate epithelial growth and reduce swelling and secretion. Bacterial resistance to silver is a concerning perspective; however, application of bio-based AgNPs may at least postpone it because the extracts used for their synthesis might have natural antimicrobial effects that can act synergistically with the nanosilver. Moreover, combined therapies based on biogenic AgNPs and known antibiotics might be even more effective than the use of only one of them.

The development of biogenic AgNPs-containing products, which are active against MDRB, finds its main obstacle in discovering a systematic, easy to reproduce, and scaled-up process for the production of the uniform nanoparticles with desirable properties that do not vary, which is extremely hard to achieve considering the biological provenience of the extracts. By the time these processes become viable, controlled, and understood, the incorporation of the biologically synthesized nanomaterials as novel biopharmaceuticals or their use as commercial products should find many opportunities in various fields.

## Figures and Tables

**Table 1:** Fungi-mediated AgNPs biosynthesis and their activity against (MDRB).

Fungus	AgNPs Size (nm)	Target MDR Microorganism	Test Type ^a^	Test Result ^b^	Reference
*Aspergillus flavus*	5–30	*E. coli*	ZI	15 ± 1.5 mm	[14]
		*S. aureus*	ZI	16 ± 2 mm	
		*M. luteus*	ZI	14 ± 1 mm	
		*P. aeruginosa*	ZI	14 ± 1.5 mm	
		*E. faecalis*	ZI	15 ± 1.5 mm	
		*A. baumanii*	ZI	15 ± 1 mm	
		*K. pneumoniae*	ZI	14 ± 0.6 mm	
		*Bacillus* spp.	ZI	15 ± 1.5 mm	
*Fusarium oxysporum NGD*	16.3–70	*Enterobacter* sp.	ZI	31 mm	[74]
		*P. aeruginosa*	ZI	20 mm	
		*K. pneumoniae*	ZI	19 mm	
		*E. coli*	ZI	2 mm	
*Trichoderma viride*	5–40	*E. coli*	ZI	16–28 mm (*)	[75]
		*S. typhi*	ZI	19–36 mm (*)	
		*S. aureus*	ZI	10–19 mm (*)	
		*M. luteus*	ZI	9–17 mm (*)	
*Aspergillus niger*	30–40	*S. aureus*	ZI	15 ± 0.23 mm	[76]
		*B. cereus*	ZI	16 ± 0.32 mm	
		*P. vulgaris*	ZI	14 ± 0.26 mm	
		*E. coli*	ZI	14 ± 0.44 mm	
		*V. cholerae*	ZI	13 ± 0.51 mm	
*Tricholoma crassum*	5–50	*E. coli* (DH5 α)	ZI	17.5 ± 0.5 (**)	[77]
		*A. tumifaciens* (LBA4404)	ZI	20.0 ± 0.5 (**)	
*Agaricus bisporus*	-	*E. coli*	ZI	14 mm	[78]
		*Klebsiella* sp.	ZI	15 mm	
		*Pseudomonas* sp.	ZI	-	
		*Enterobacter* sp.	ZI	18 mm	
		*Proteus* sp.	ZI	20 mm	
		*S. aureus*	ZI	17 mm	
		*S. typhi*	ZI	22 mm	
		*S. paratyphi*	ZI	17 mm	
*Aspergillus clavatus*	550–650 (AFM)	*S. aureus*	ZI	20.5 mm	[79]
		*S. epidermidis*	ZI	19 mm	
*Penicilium polonicum*	10–15	*A. baumanii*	MIC, MBC, ZI	15.62 μg mL^−1^ (MIC), 31.24 μg mL^−1^ (MBC), 21.2 ± 0.4 mm (ZI)	[73]
*Cryphonectria* sp.	30–70	*S. aureus* (ATCC-25923)	ZI	16 ± 0.69 mm	[80]
		*S. typhi* (ATCC-51812)	ZI	12 ± 0.29 mm	
		*E. coli* (ATCC-39403)	ZI	13 ± 1.54 mm	
*Rhizoppus* spp.	27–50	*E. coli*	ZI	15–22 mm (***)	[81]
*Fusarium oxysporum*	77.68	*S. aureus* (MRSA 101)	MIC, MBC	250 μM (MIC), 500 μM (MBC)	[70]
		*S. aureus* (MRSA 107)	MIC, MBC	250 μM (MIC), 500 μM (MBC)	
		*E. coli* (ESBL 167)	MIC, MBC	125 μM (MIC), 125 μM (MBC)	
		*E. coli* (ESBL 169)	MIC, MBC	125 μM (MIC), 125 μM (MBC)	
		*E. coli* (ESBL 176)	MIC, MBC	125 μM (MIC), 125 μM (MBC)	
		*E. coli* (ESBL 192)	MIC, MBC	125 μM (MIC), 125 μM (MBC)	
		*E. coli* (KPC 131)	MIC, MBC	125 μM (MIC), 125 μM (MBC)	
		*E. coli* (KPC 133)	MIC, MBC	125 μM (MIC), 125 μM (MBC)	
		*A. baumannii* (CR 01)	MIC, MBC	125 μM (MIC), 125 μM (MBC)	
*Aspergillus flavus*	5–30	*E. coli*	ZI	15 ± 1.5 mm	[14]
		*S. aureus*	ZI	16 ± 2 mm	
		*M. luteus*	ZI	14 ± 1 mm	
		*P. aeruginosa*	ZI	14 ± 1.5 mm	
		*E. faecalis*	ZI	15 ± 1.5 mm	
		*A. baumannii*	ZI	15 ± 1 mm	
		*K. pneumoniae*	ZI	14 ± 0.6 mm	
		*Bacillus* spp.	ZI	15 ± 1.5 mm	
*Macrophomina phaseolina*	5–40	*E. coli* (DH5α-MDR)	ZI	3.0 ± 0.2 mm (**)	[72]
		*A. tumefaciens* (LBA4404-MDR)	ZI	3.3 ± 0.2 mm (**)	

^a^ ZI = zone of inhibition; MIC = Minimum Inhibitory Concentration; MBC = Minimum Bactericidal Concentration.; ^b^ For tests in which more than one concentration of AgNPs was used, the best results are shown; (*) Values related to a synergistic effect with distinct antibiotics; (**) Values estimated from graphs; (***) More than one bacterial isolate was used.

**Table 2:** Bacteria-mediated AgNPs biosynthesis and their activity against MDRB.

Bacteria	AgNPs Size (nm)	Target MDR Microorganism	Test Type ^a^	Test Result ^b^	Reference
*Streptomyces*	20–70	*K. pneumoniae* (ATCC 100603)	MIC	4 μg mL^−1^	[92]
		*K. pneumoniae*	MIC	1.4 μg mL^−1^	
		*E. coli*	MIC	2 μg mL^−1^	
		*Citrobacter*	MIC	2 μg mL^−1^	
*Bacillus* sp.	14–42	*S. epidermidis strain 73* (pus)	ZI	15 mm	[93]
		*S. epidermidis strain 145* (catheter tips)	ZI	19 mm	
		*S. epidermidis strain 152* (blood)	ZI	19 mm	
		*S. aureus* (MTCC 87)	ZI	18 mm	
		*S. typhi*	ZI	13 mm	
		*S. paratyphi*	ZI	15 mm	
		*V. cholerae* (MTCC 3906)	ZI	18 mm	
*Bacillus cereus*	24–46	*E. coli*	MIC, ZI	6.25 μg mL^−1^ (MIC), 16 ± 1 mm (ZI)	[94]
		*S. aureus*	MIC, ZI	12.5 μg mL^−1^ (MIC), 14 ± 1 (ZI)	
		*K. pneumoniae*	MIC, ZI	>3.12 μg mL^−1^ (MIC), 17 ± 1 mm (ZI)	
		*P. aeruginosa*	MIC, ZI	3.12 μg mL^−1^ (MIC), 23 ± 1 mm (ZI)	
*Bacillus safensis* (LAU 13)	5–95	*E. coli*	ZI	11–19 mm	[95]
		*K. granulomatis*	ZI	11–19 mm	
		*P. vulgaris*	ZI	11–19 mm	
		*P. aeruginosa*	ZI	11–19 mm	
		*S. aureus*	ZI	11–19 mm	
*Aeromonas* sp. THG-FG1.2	8–16	*B. cereus* (ATCC 14579)	ZI	13.5 ± 0.5 mm	[88]
		*B. subtilis* (KACC 14741)	ZI	13 ± 1.0 mm	
		*S. aureus* (ATCC 6538)	ZI	15.5 ± 0.5 mm	
		*E. coli* (ATCC 10798)	ZI	13 ± 0.2 mm	
		*P. aeruginosa* (ATCC 6538)	ZI	16 ± 0.1 mm	
		*V. parahaemolyticus* (ATCC 33844)	ZI	16 ± 0.1 mm	
		*S. enterica* (ATCC 13076)	ZI	11 ± 0.2 mm	
		*C. albicans* (KACC 30062)	ZI	20 ± 0.1 mm	
		*C. tropicalis* (KCTC 7909)	ZI	15 ± 0.5 mm	
*Bacillus thuringiensis*	15	*E. coli*	ZI	12 ± 1 mm (*)	[96]
		*P. aeruginosa*	ZI	16 ± 1 mm (*)	
		*S. aureus*	ZI	9 ± 1 mm (*)	
*Anabaena diololum*	10–50	*K. pneumoniae* DF12SA (HQ114261)	ZI	36 ± 0.82 mm	[97]
	10–50	*E. coli* DF39TA (HQ163793)	ZI	33 ± 1.63 mm	
	10–50	*S. aureus* DF8TA (JN642261)	ZI	34 ± 0.81 mm	
*Streptomyces* sp. GUT 21	23–48	*E. coli* (MTCC 9537)	MIC, ZI	14 μg mL^−1^ (MIC), 27.05 ± 3.20 mm (ZI)	[89]
		*K. pneumoniae* (MTCC 109)	MIC, ZI	12 μg mL^−1^ (MIC), 28.50 ± 2.60 mm (ZI)	
		*S. aureus* (MTCC 96)	MIC, ZI	15 μg mL^−1^ (MIC), 24.25 ± 2.09 mm (ZI)	
		*P. aeruginosa* (MTCC 1688)	MIC, ZI	10 μg mL^−1^ (MIC), 10.05 ± 3.60 mm (ZI)	
*Bacillus megaterium*	80–98.56 (AFM)	*S. pneumoniae*	ZI	21 mm	[98]
		*S. typhi*	ZI	18 mm	
*Xanthomonas* spp.	5–40	*P. aeruginosa*	ZI	10.0 ± 1.0 mm	[91]
		*baumannii*	ZI	10.6 ± 0.6 mm	
*Sinomonas mesophila* MPKL 26	4–50	*S. aureus*	ZI	12 mm	[90]
*Bacillus flexus*	12–65	*E. coli*	ZI	11.55 mm	[99]
		*P. aeruginosa*	ZI	11.05 mm	
		*S. pyogenes*	ZI	11.65 mm	
		*subtilis*	ZI	11.55 mm	
*Bacillus brevis* (NCIM 2533)	41–68	*S. aureus*	ZI	19 mm	[100]
		*S. typhi*	ZI	7.5 mm	

^a^ ZI = zone of inhibition; MIC = Minimum Inhibitory Concentration; MBC = Minimum Bactericidal Concentration; ^b^ For tests in which more than one concentration of AgNPs was used, the best results are shown; (*) Values estimated from graphs.

**Table 3:** Plant-mediated AgNPs biosynthesis and their activity against MDRB.

Plant	Part	AgNPs Size (nm)	Target MDR Microorganism	Test Type ^a^	Test Result ^b^	Reference
Olive	leaf	20–25	*S. aureus*	ZI	2.4 ± 0.2 cm (*)	[114]
			*P. aeruginosa*	ZI	2.4 ± 0.2 cm (*)	
			*E. coli*	ZI	1.8 ± 0.2 cm (*)	
*Phyllanthus amarus*	Whole plant	24 ± 8	*P. aeruginosa*	MIC, ZI	6.25–12.5 μg mL^−1^ (MIC), 10 ± 0.53 to 21 ± 0.11 mm (ZI)	[115]
*Corchorus capsularis*	leaf	5–45	*P. aeruginosa*	ZI	17 mm	[116]
			*S. aureus*	ZI	21 mm	
			Coagulase negative staphylococci	ZI	20 mm	
*Tribulus terrestris*	fruit	16–28	*S. pyogens*	ZI	10 mm	[117]
			*E. coli*	ZI	10.75 mm	
			*P. aeruginosa*	ZI	9.25 mm	
			*B. subtilis*	ZI	9.25 mm	
			*S. aureus*	ZI	9.75 mm	
*Garcinia mangostana*	leaf	35	*E. coli*	ZI	15 mm	[118]
			*S. aureus*	ZI	20 mm	
*Ricinus communis*	leaf	29.18 (X-ray diffraction)	*B. fusiformis*	ZI	2.90 cm	[119]
			*E.coli*	ZI	2.89 cm	
*Caesalpinia coriaria*	leaf	40–52	*E. coli*	ZI	12.0 ± 0.50 mm	[109]
			*P. aeruginosa*	ZI	18.3 ± 0.80 mm	
			*K. pneumonia*	ZI	14.6 ± 1.20 mm	
			*S. aureus*	ZI	10.3 ± 1.20 mm	
		78–98	*E. coli*	ZI	9.6 ± 0.80 mm	[109]
			*P. aeruginosa*	ZI	18.3 ± 1.20 mm	
			*K. pneumonia*	ZI	13.3 ± 0.30 mm	
			*S. aureus*	ZI	11.0 ± 0.00 mm	
*Mimusops elengi*	leaf	55–83	*K. pneumoniae*	ZI	18 mm	[120]
			*S. aureus*	ZI	10 mm	
			*M. luteus*	ZI	11 mm	
*Ocimum gratissimum*	leaf	16 ± 2	*E. coli* (*MC-2*)	MIC, MBC, ZI	4 μg mL^−1^ (MIC), 8 μg mL^−1^ (MBC), 12 ± 0.6 mm (ZI)	[112]
			*S. aureus* (*MMC-20*)	MIC, MBC, ZI	8 μg mL^−1^ (MIC), 16 μg mL^−1^ (MBC), 16 ± 1.0 mm (ZI)	
*Hydrocotyle sibthorpioides*	Whole plant	13.37 ± 10	*K. pneumonia*	ZI	3.0 ± 0.17 mm	[121]
			*P. aeruginosa*	ZI	2.7 ± 0.32 mm	
			*S. aureus*	ZI	3.6 ± 0.57 mm	
*Vaccinium corymbosum*	leaf	10–30	*E. coli* (ATCC 25922)	MIC, MBC, ZI	11.22 ± 0.29 mm	[122]
			*S. aureus* (ATCC 25923)	MIC, MBC, ZI	13.1 ± 1.1 mm	
			*P. aeruginosa* (ATCC 27853)	MIC, MBC, ZI	11.6 ± 0.32 mm	
			*B. subtilis* (ATCC 21332)	MIC, MBC, ZI	12.4 ± 0.40 mm	
*Prosopis farcta*	leaf	10.8 ± 3.54	*S. aureus* (PTCC 1431)	ZI	9.5 mm	[123]
			*B. subtilis* (PTCC 1420)	ZI	9 mm	
			*E. coli* (PTCC 1399)	ZI	9.5 mm	
			*P. aeruginosa* (PTCC 1074)	ZI	9.5 mm	
*Sesbania gradiflora*	leaf	10–25	*S. enterica*	ZI	15.67 ± 0.09 mm	[124]
			*S. aureus*	ZI	10.54 ± 0.23 mm	
*Solanum nigrum*	leaf	20	*K. pneumoniae*	ZI	21.5 mm	[110]
			*P. aeruginosa*	ZI	21.3 mm	
			*S. epidermidis*	ZI	19.6 mm	
			*E. coli*	ZI	15.3 mm	
			*P. vulgaris*	ZI	13.3 mm	
			*S. aureus*	ZI	9.6 mm	
*Cissus quadrangularis*	leaf	15–23 (**)	*S. pyogens*	MIC, ZI	4 μg mL^−1^ (MIC), 7.77 ± 0.25 mm (ZI)	[113]
			*S. aureus*	MIC, ZI	3 μg mL^−1^ (MIC), 8.83 ± 0.26 mm (ZI)	
			*E. coli*	MIC, ZI	5 μg mL^−1^ (MIC), 7.9 ± 0.31 mm (ZI)	
			*P. vulgaris*	MIC, ZI	7 μg mL^−1^ (MIC), 8.4 ± 0.40 mm (ZI)	
*Cola nitida*	pod	12–80	*E. coli*	ZI	19 ± 0.9 mm	[125]
			*K. granulomatis*	ZI	11 ± 0.8 mm	
			*P. aeruginosa*	ZI	28 ± 0.1 mm	
*Strychnos potatorum*	leaf	28	*S. aureus*	ZI	8 mm	[126]
			*K. pneumoniae*	ZI	10 mm	
*Alstonia scholaris*	leaf	80	*E. coli*	ZI	10.0 ± 2.8 mm	[111]
			*P. aeruginosa*	ZI	8.0 ± 1.4 mm	
			*K. pneumoniae*	ZI	11.0 ± 1.0 mm	
			*S. aureus*	ZI	10.0 ± 3.0 mm	
			*P. vulgaris*	ZI	8.3 ± 0.6 mm	
			*S. epidermidis*	ZI	10.6 ± 1.2 mm	
*Andrographis paniculata*	leaf	70	*E. coli*	ZI	8.0 ± 1.4 mm	[111]
			*P. aeruginosa*	ZI	6.7 ± 0.7 mm	
			*K. pneumoniae*	ZI	9.3 ± 0.6 mm	
			*S. aureus*	ZI	8.0 ± 1.0 mm	
			*P. vulgaris*	ZI	8.3 ± 0.6 mm	
			*S. epidermidis*	ZI	9.0 ± 1.0 mm	
*Aegle marmelos*	leaf	70	*E. coli*	ZI	11.0 ± 2.8 mm	[111]
			*P. aeruginosa*	ZI	9.0 ± 1.4 mm	
			*K. pneumoniae*	ZI	9.3 ± 1.6 mm	
			*S. aureus*	ZI	9.7 ± 1.5 mm	
			*P. vulgaris*	ZI	9.7 ± 0.6 mm	
			*S. epidermidis*	ZI	8.0 ± 1.0 mm	
*Centella asiatica*	leaf	90	*E. coli*	ZI	12.7 ± 0.7 mm	[111]
			*P. aeruginosa*	ZI	8.0 ± 1.4 mm	
			*K. pneumoniae*	ZI	12.0 ± 1.0 mm	
			*S. aureus*	ZI	13.0 ± 2.0 mm	
			*P. vulgaris*	ZI	9.7 ± 0.6 mm	
			*S. epidermidis*	ZI	14.0 ± 1.0 mm	
*Eclipta prostrata*	leaf	70	*E. coli*	ZI	10.0 ± 4.0 mm	[111]
			*P. aeruginosa*	ZI	8.3 ± 2.5 mm	
			*K. pneumoniae*	ZI	10.0 ± 5.2 mm	
			*S. aureus*	ZI	12.6 ± 4.9 mm	
			*P. vulgaris*	ZI	6.6 ± 0.5 mm	
			*S. epidermidis*	ZI	8.0 ± 0.0 mm	
*Moringa oleifera*	leaf	50	*E. coli*	ZI	7.7 ± 0.6 mm	[111]
			*P. aeruginosa*	ZI	8.0 ± 1.7 mm	
			*K. pneumoniae*	ZI	7.0 ± 1.0 mm	
			*S. aureus*	ZI	9.0 ± 2.6 mm	
			*P. vulgaris*	ZI	7.0 ± 2.0 mm	
			*S. epidermidis*	ZI	7.0 ± 0.0 mm	
*Thespesia populnea*	bark	70	*E. coli*	ZI	9.0 ± 1.7 mm	[111]
			*P. aeruginosa*	ZI	10.3 ± 2.1 mm	
			*K. pneumoniae*	ZI	11.3 ± 1.2 mm	
			*S. aureus*	ZI	9.3 ± 2.4 mm	
			*P. vulgaris*	ZI	8.6 ± 1.2 mm	
			*S. epidermidis*	ZI	8.6 ± 0.7 mm	
*Terminalia arjuna*	bark	70	*E. coli*	ZI	8.0 ± 0.7 mm	[111]
			*P. aeruginosa*	ZI	9.0 ± 2.0 mm	
			*K. pneumoniae*	ZI	14.0 ± 1.0 mm	
			*S. aureus*	ZI	12.7 ± 1.1 mm	
			*P. vulgaris*	ZI	8.3 ± 0.6 mm	
			*S. epidermidis*	ZI	9.0 ± 2.0 mm	
*Plumbago zeylanica*	Root bark	90	*E. coli*	ZI	8.0 ± 1.4 mm	[111]
			*P. aeruginosa*	ZI	14.7 ± 0.7 mm	
			*K. pneumoniae*	ZI	8.3 ± 0.8 mm	
			*S. aureus*	ZI	7.7 ± 0.6 mm	
			*P. vulgaris*	ZI	8.3 ± 0.6 mm	
			*S. epidermidis*	ZI	8.0 ± 1.0 mm	
*Semecarpus anacardium*	nuts	60	*E. coli*	ZI	10.0 ± 2.0 mm	[111]
			*P. aeruginosa*	ZI	9.3 ± 1.5 mm	
			*K. pneumoniae*	ZI	10.0 ± 1.0 mm	
			*S. aureus*	ZI	7.7 ± 1.1 mm	
			*P. vulgaris*	ZI	8.3 ± 0.6 mm	
			*S. epidermidis*	ZI	9.3 ± 1.5 mm	
*Mukia scabrella*	leaf	18–21	*Acinetobacter* sp.	ZI	22 mm	[127]
			*K. pneumoniae*	ZI	19 mm	
			*P. aeruginosa*	ZI	20 mm	
*Phyllanthus amarus*	Whole plant	24 ± 8	*P. aeruginosa* (***)	MIC, ZI	6.25–12.5 μg mL^−1^ (MIC), 21 ± 0.11 mm (ZI)	[115]
*Ricinodendron heudelotti*	Seed kernel	89.0	*E. coli*	MIC, MBC	1.68 μg mL^−1^ (MIC), 6.75 μg mL^−1^ (MBC)	[128]
*Gnetum bucholzianum*	leaf	67.4	*E. coli*	MIC, MBC	1.687 μg mL^−1^ (MIC), 1.687 μg mL^−1^ (MBC)	[129]
*Megaphrynium macrostachyum*	leaf	33.7 (Ag), 44.2 (AgCl)	*E. coli*	MIC, MBC	0.515 μg mL^−1^ (MIC), 4.12 μg mL^−1^ (MBC)	[129]
*Corchorus olitorus*	leaf	30.0 (nm), 37.9 (AgCl)	*E. coli*	MIC, MBC	8.25 μg mL^−1^ (MIC), 16.5 μg mL^−1^ (MBC)	[129]
*Ipomoea batatas*	leaf	67.3 (Ag), 37.9 (AgCl)	*E. coli*	MIC, MBC	5.3 μg mL^−1^ (MIC), 5.3 μg mL^−1^ (MBC)	[129]
*Areca catechu*	leaf	22–40	*E. coli*	ZI	20 mm	[129]
			*P. aeruginosa*	ZI	24 mm	
			*S. typhi*	ZI	19 mm	
			*P. vulgaris*	ZI	23 mm	
			*K. pneumoniae*	ZI	26 mm	
Cocoa	bean	8.96–54.22	*S. aureus*	ZI	12 mm (*)	[130]
			*K. pneumoniae* (wound)	ZI	12 mm (*)	
			*K. pneumoniae* (urine)	ZI	13 mm (*)	
			*E. coli*	ZI	14 mm (*)	
Cocoa	Pod husk	4–32	*K. pneumoniae*	ZI	10–14 mm	[131]
			*E. coli*	ZI	10–14 mm	
*Phomis bracteosa*	Whole plant	22.41	*E. coli* (ATCC 15224)	ZI	13.2 ± 0.12	[108]
			*S. aureus* (ATCC 6538)	ZI	11.1 ± 0.10	
			*K. pneumoniae* (ATCC 4619)	ZI	10.3 ± 0.11	
*Momordica cymbalaria*	fruit	15.5	*E. coli*	ZI	24.0 ± 1.0	[132]
			*M. luteus*	ZI	20.0 ± 1.4	
			*B. cereus*	ZI	22.0 ± 1.0	
			*K. pneumoniae*	ZI	26.0 ± 1.4	
			*S. pneumoniae*	ZI	26.0 ± 1.7	
*Astragalus membranaceus*	root	65.08	*S. aureus* (MRSA)	MIC, ZI	0.063 mg mL^−1^ (MIC), 12.83 ± 1.04 mm (ZI)	[107]
			*S. epidermidis* (MRSE)	MIC, ZI	0.063 mg mL^−1^ (MIC), 12.33 ± 0.29 mm (ZI)	
			*P. aeruginosa*	MIC, ZI	0.032 mg mL^−1^ (MIC), 15.17 ± 0.76 mm (ZI)	
			*E. coli*	MIC, ZI	0.032 mg mL^−1^ (MIC), 14.67 ± 0.76 mm (ZI)	

^a^ ZI = zone of inhibition; MIC = Minimum Inhibitory Concentration; MBC = Minimum Bactericidal Concentration; ^b^ For tests in which more than one concentration of AgNPs was used, the best results are shown; (*) Values estimated from graphs; (**) Silver chloride nanoparticles; (***) 15 strains were tested

**Table 4:** Patents of AgNPs-based products tested against resistant bacterial strains.

Patent Number	Application	Resistant Bacteria	Reference
WO2006074117A2	Hydrogel	*E. cloacae*, *K. pneumoniae*, *E. coli*, *P. aeruginosa*, *A. Acinetobacter*	[170]
WO2018010403A1	Pharmaceuticals	*E. cloacae*, *K. pneumoniae*, *E. coli*, *P. aeruginosa*, *A. Acinetobacter*	[171]
US20100003296A1	Textiles	Methicillin-resistant *S. aureus* (MRSA)	[172]
KR200384433Y1	Apron, perfume	Methicillin-resistant *S. aureus* (MRSA)	[173]
KR100933736B1	Detergent additive	*E. coli*	[174]
CN105412940A	General	Vancomycin-resistant *Enterococcus faecalis*	[175]
WO2005120173A2	General	*P. aeruginosa*	[176]
US7135195B2	General	Methicillin-resistant *S. aureus* (MRSA)	[177]
